# The role of fetal heart rate in first trimester sonograms in prediction of fetal sex: a systematic review and meta-analysis

**DOI:** 10.1186/s12884-023-05908-8

**Published:** 2023-08-12

**Authors:** Shadi Nouri, Mohammad Hassan Kalantar, Fatemeh Safi, Amir Almasi-Hashiani

**Affiliations:** 1 Department of Radiology, School of Medicine Arak, University of Medical Sciences, Arak, Iran; 2 Student Research Committee, Arak University of Medical Sciences, Arak, Iran; 3 Department of Epidemiology, School of Health, Arak University of Medical Sciences, Arak, Iran; 4 Traditional and Complementary Medicine Research Center (TCMRC), Arak University of Medical Sciences, Arak, Iran

**Keywords:** Fetus, Gender, Fetal heart rate, Meta-analysis

## Abstract

**Background:**

Early fetal sex determination is worthy of providing alertness about possible x-linked disorders, as well as predicting sex-related pregnancy complications and outcomes. Satisfying the curiosity of parents is another advantage. In this way, several studies have been performed which have shown conflicting results.

**Aim:**

We planned a systematic review for identifying any plausible role of Fetal Heart Rate (FHR) for early predicting fetal sex during the first trimester of non-complicated pregnancies.

**Methods:**

This is a meta-analysis in which PubMed and Scopus databases were searched using different related keywords to find similar articles up to December 2022. Then the articles were screened to find eligible articles and finally, the articles entered in the meta-analysis were analyzed using Stata software (Stata Corp, College Station, TX). Standardized mean difference (SMD) and their 95% confidence interval (CI) were estimated.

**Results:**

A total of 223 articles were evaluated and five articles were included in the meta-analysis. The results showed that there is a significant heterogeneity between the articles (*p* = 0.012, I-squared = 69.0%). The results of meta-analysis with a random model showed that there is no significant difference between male and female genders in terms of mean FHR (SMD = 0.04, 95%CI = -0.09–0.16, Z = 0.59, *p* = 0.553).

**Conclusion:**

This systematic review and meta-analysis showed that even though male fetuses show faster FHR but such sex-related difference is minimal. Therefore, first-trimester FHR is not a reliable predictive test for fetal sex determination. Further studies are recommended to achieve a more precise conclusion.

**Trial registration:**

PROSPERO: CRD42023418291.

## Introduction

Fetal sex determination is one of the integral parts of second-trimester sonograms, which is achievable by direct visualization of the fetus’s external genitalia [1] but early detection is worthful, because higher rate of complicated pregnancies (by gestational diabetes mellitus and pre-eclampsia) and also more probability of need for cesarean section, have been reported for male fetuses. Awareness about the fetus’s sex helps physicians to be prepared for diagnosis and management of possible sex-related disorders as well [2–4]. Satisfying the parents' curiosity about the fetus gender is another advantage.

Although laboratory exams including invasive methods like amniocentesis, chorionic villous sampling [5, 6], and non-invasive cell-free DNA analysis [7] are highly accurate tests for this purpose, many sonographic methods have been also suggested and used worldwide. Sonographic sex determination in the first trimester is provided by several suggested methods such as measurement of the angle between the genital tubercle and horizontal line tangent to the lumbosacral skin (on sagittal sonograms), which has various reported accuracy from 56% [8] to 90.1% [9] for both and 97% for female sex [10]. Anogenital distance measurement is also another recommended technique that differs between populations and needs nomograms [11]. Both methods need an acceptable resolution of sonographic equipment.

Sex prediction based on Fetal Heart Rate (FHR) which can be achieved even by old sonographic devices, is another suggested simple method. Several studies have investigated the relationship between perinatal fetal heart rate and fetal gender. Results are inconsistent as the study by Hall et al. in 1993 showed female fetuses have FHR greater than 140 bpm [12] but most of the others exhibited no significant difference [3, 4, 13–15].

A couple of studies also evaluated this relationship in the first trimester which continued to show different but non-significant results [16–19]. FHR is influenced by many factors like uterine contractions, fetal breathing and movement [20], or exogenous glucocorticoid administration in the third trimester and peripartum period [21]. Also, fluctuations in FHR in the first trimester are the least [22]. Considering such conflicting results, we planned a systematic review for identifying any plausible role of FHR for early predicting fetal sex during the first trimester of non-complicated pregnancies.

## Methods

### Study design

This study is a systematic review and meta-analysis which was performed according to the standard guideline of “*Preferred Reporting Items for Systematic Reviews and Meta-Analyses* (PRISMA)” [23]. This study was registered with the International Prospective Register of Systematic Reviews (PROSPERO) available at: https://www.crd.york.ac.uk/prospero/, CRD42023418291.

### Search strategy

The search strategy was such that PubMed and Scopus databases were searched using different keywords to find similar articles. In PubMed, various tags including Medical Subject Heading (MeSH), all fields (all), and text words (tw) were used to search for different keywords. Keywords searched in PubMed were adapted for searching in Scopus. The last search of databases was done on April 2023. The keywords used in the PubMed search included: "Heart Rate, Fetal"[Mesh], "Fetal Heart Rate"[tw], "Fetal sex"[tw], "Fetus sex"[tw], "Fetal sex"[all] and "Fetal Gender"[all]. All searches were conducted by one author (AAH). In addition to the mentioned databases, to find gray literature, Google Scholar was also searched and the title of found studies were screened up to page 20 (200 studies) of Google Scholar.

### Inclusion and exclusion criteria

In this meta-analysis, studies with the following characteristics were included in the analysis. 1) Articles whose study population is first-trimester pregnancies that underwent ultrasound and the results of the fetal heart rate in the first trimester were compared with the gender of the fetus that was determined in the second trimester. 2) Studies with full text in English, 3) and in terms of study design, all cross-sectional, case–control, and cohort articles can be included in this review. The study does not include case reports, case series, commentary, and letter to the editor. There is no time limit in search and screening. Finally, all the studies that reported the necessary data, including the mean and standard deviation of the fetal heart rate, along with the sample size by gender, were included in the analysis.

### Study selection

The articles retrieved from the databases were entered into Endnote X8 software. First, duplicate studies were removed, and then the studies were screened based on the study title and abstract, and the studies that met the inclusion criteria were retained for further evaluations, and the rest were removed. After that, studies were screened using the full text of the remaining articles, and studies that did not meet the inclusion criteria were excluded. Finally, the required data were extracted from the remaining articles. Screening of articles was done by two researchers independently (SN and MHK), and in cases where two authors did not agree, a decision was made regarding the removal or keeping of the articles with the consultation of another author (FS).

### Data extraction

From the remaining articles that were candidates for meta-analysis, the required data were extracted by two authors independently (SN and MHK), and in cases where there was a disagreement between the authors, decisions were made in consultation with the other author (AAH). The data extracted from the studies included: the name of the first author, the year of publication of the study, the total sample size as well as the sample size by gender of the fetus, the mean age of mothers, the mean and standard deviation of the FHR by gender, country of study and study quality score.

### Risk of bias in individual studies

To assess the risk of bias or study quality, an assessment of the remaining studies was performed using the Newcastle–Ottawa Assessment Scale (NOS). This scale is designed for case–control, cohort, and cross-sectional studies. Using this checklist, articles are classified into three groups based on study quality, including articles with a score of 6 to 9 (low risk), articles with a score of 3 to 6 (moderate risk), and articles with a score of less than 3 (high risk). The items in this checklist include items related to the selection of participants, comparison, and exposure.

### Statistical analysis

Table and forest plot were used to report the results. “*metan*” package was used in Stata software version 13 for data analysis (Stata Corp, College Station, TX, USA). To check the heterogeneity among studies, in addition to the I-square statistic, the chi-square test was used, and in cases where there was significant heterogeneity between studies, the random-effects model was used to combine the results. Since the aim of the study was to compare the mean of a quantitative variable (FHR) between two groups (male and female fetuses), the effect size reported in the plot and the text is the standardized mean difference (SMD) (with 95% confidence interval (CI)). Since the number of studies was relatively small, both the graphical method (funnel plot) and statistical method of Egger tests were used to investigate the possibility of publication bias, and based on the results of these methods, a decision was made regarding publication bias, and due to the publication bias, the trim and filled method was used. Finally, sensitivity analysis was also performed to check the impact of each study on the overall estimated SMD.

## Results

### Study selection and study characteristics

**Fig. 1:**
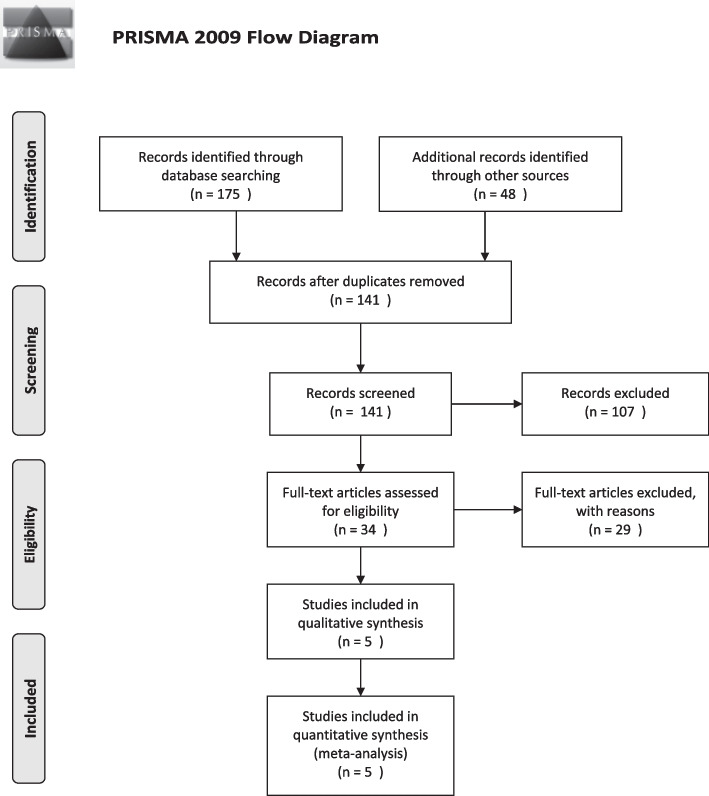
Flow diagram of the literature search for studies included in meta-analysis

The steps of searching and screening the retrieved studies from the databases are shown in Fig. 1. In total, 175 articles were found from two databases, PubMed and Scopus (53 articles from PubMed and 122 articles from Scopus). In addition, 48 articles were found by Google Scholar, and a total of 223 articles were evaluated. After that, duplicate articles (82 articles) were removed and the remaining 141 articles were evaluated in terms of title and abstract, and 107 articles did not meet the inclusion criteria and were excluded. In the next step, the full text of the remaining 34 articles was screened, and as a result, 29 articles were excluded due to lack of required data or lack of inclusion criteria, and five [16–19, 24] articles were included in the meta-analysis.

The oldest article was from 1989 in the USA [17] and the most recent was from 2022 in Nigeria [24]. Out of these 5 articles, 3 studies were designed in the USA [16, 17, 19], one study in Iran [18], and one study in Nigeria [24]. More details about the articles are shown in Table 1.

**Table 1:** A summary of the included study characteristics

Author	Year	Country	Sample size	Maternal age	NOS score	Mean of FHR in Male	Mean of FHR in female	SD of FHR in male	SD of FHR in female	Male number	Female number
DuBose T.J. [17]	1989	USA	365	-	7	151.1	150.2	13.2	14.3	178	187
McKenna D.S. [19]	2005	USA	477	28.4	7	154.9	151.7	22.8	22.7	233	244
Bracero L.A. [16]	2015	USA	655	24.2	7	167.3	167	10.1	9.1	323	332
Keshavarz E. [18]	2018	Iran	374	23.1	7	156.21	154.94	8.42	7.71	198	176
Oloyede O.A. [24]	2022	Nigeria	2437	-	7	163.2	165.4	17.1	18.2	1398	1039

### Risk of bias within studies

The standard Newcastle–Ottawa scale was used to evaluate the quality of each article. Based on this scale, all articles were classified as low risk.

### Quantitative data synthesis and heterogeneity of studies

A total of 5 articles [16–19, 24] were included in the quantitative meta-analysis. At first, the heterogeneity between the articles was checked and the results showed that there is significant heterogeneity between the articles (Heterogeneity chi-squared = 12.9, (d.f. = 4), p = 0.012, I-squared (variation in SMD attributable to heterogeneity) = 69.0% and estimate of between-study variance Tau-squared = 0.013), therefore, based on the Cochrane guidelines, the random-effects model was used to combine the results.

**Fig. 2:**
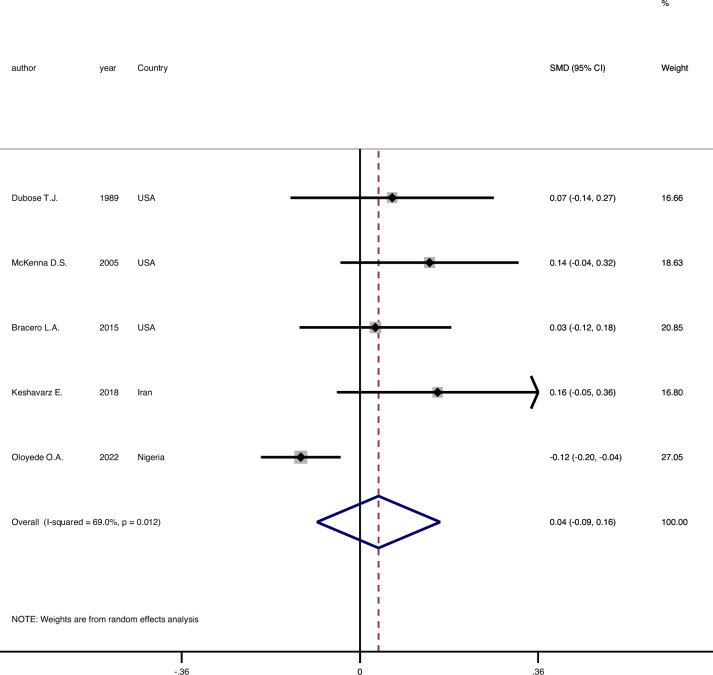
Forest plot to compare the FHR means between genders

As it was presented in Fig. 2, the results of the meta-analysis with a random model showed that there is no significant difference between male and female genders in terms of mean FHR (SMD = 0.04, 95%CI = -0.09–0.16, Z = 0.59, p = 0.553).

### Risk of bias across studies

**Fig. 3:**
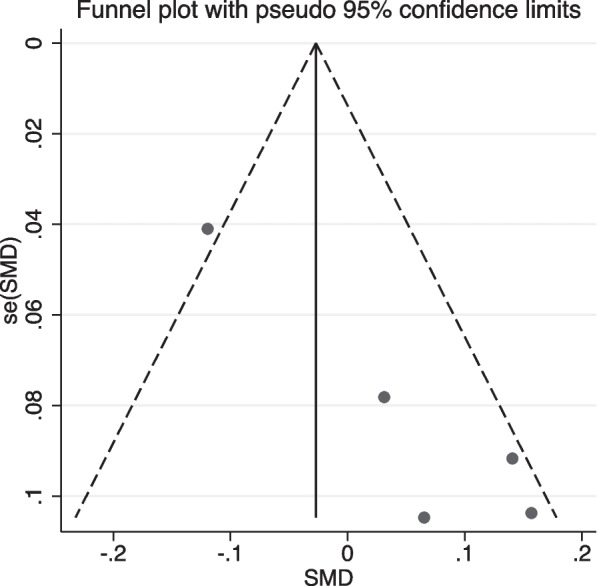
Funnel plot to check the publication bias

Investigation of the possibility of publication bias in the articles was done and the Funnel plot (Fig. 3) showed a significant asymmetry in the distribution of the articles. In addition, the Egger test results were also significant in favor of publication bias (t = 6.37, p = 008).

Due to the existence of publication bias, the trim and fill method was performed and the meta-analysis was repeated with a random-effects model (SMD = -0.076, 95%CI = -0.198–0.045, Z = -1.23, p = 0.219) and the estimation made did not change significantly.

### Sensitivity analysis

For further investigations, a sensitivity analysis was also performed, in which each of the articles was excluded from the meta-analysis, and the meta-analysis was repeated with the remaining four articles. Different results were observed following the sensitivity analysis only when Oloyede et al. study [24] was removed. After this study was removed, the results of the meta-analysis became significant and the mean FHR in male fetuses was significantly higher than that of female fetuses (SMD = 0.091, 95%CI = 0.001–0.181).

## Discussion

This systematic review and meta-analysis showed that even though male fetuses show faster FHR but such sex-related difference is minimal. Therefore, first-trimester FHR is not a reliable predictive test for fetal sex determination.

Fetal sex determination is necessary in many ways. Some fetal disorders are x-linked and needed to be assessed during pregnancy [25]. There is also a higher risk of gestational and perinatal complications for male fetuses [1–4]. Hence early sex determination seems a necessary issue that helps clinicians to be aware of and prepared for any possible complications. There is a need for a safe, non-invasive, widely available method for such a purpose. Ultrasound is a relatively safe way of obtaining images without provoking harmful effects on the fetus [26]. So, sex determination based on ultrasound has been a rational issue for years. Knowledge about the different cardiovascular status of adults related to gender [27, 28], encouraged researchers to investigate such sex-related variation in the fetal period.

Studies of fetal cardiac function and fetoplacental circulation during the second and third trimesters of pregnancy revealed higher preload and lower afterload in male fetuses [29–31]. Several research projects have also been conducted during the second and third trimesters, to assess the effectiveness of FHR in this area [12, 32–34], but the results were conflicting, therefore to address the presence of any sex-related difference in FHR, we planned a systematic review of articles and considering the potential impact of confounders in the third trimester and peripartum period, we scheduled the review on articles which had been performed in the first trimester.

In this meta-analysis, we evaluated 5 peer-reviewed published articles with a total population number of 4308 fetuses. Studies by Mckenna et al. [19], DuBose et al. [17], and Keshavarz et al. [18] disclosed that despite faster FHR in male fetuses versus females, this difference is non-significant. In contrast, Oloyede et al. [24] implied that female fetuses have faster FHR but similarly, this variety is not statistically significant. Bracero et al. [16] showed no sex-related differences in FHR.

In assessing heterogeneity across studies, we faced a moderate degree of statistical heterogeneity that possibly could be related to different countries of origin (ethnic), mother’s age, gestational age, different sonographers experience, and accuracy of ultrasound machines. So, we utilized the random- Effects model instead of the fixed-effects model. In this line, the pooled estimate of the results exhibits no statistically significant difference in mean FHR between genders. The results of this meta-analysis showed that the mean FHR is similar between boys and girls. Therefore, it is not possible to predict the gender of the fetus by FHR.

Thyroid hormone, glucocorticoids, and catecholamines are among the most important hormones which take part in the process of cardiac development, similarly in both sexes [34–39]. What seems different is how sex hormones contribute to the process. Testosterone inhibits the function and production of vasodilators [40–42] leading to an increase in blood pressure [43] and preload [29].

Fetal heart variability is also different among genders so female fetuses exhibit greater heart rate dynamics in early gestational periods, suggesting that maturation of the cardiovascular system which is impressed by the autonomic nervous system, occurs earlier than that of males [5]. One might expect similar results of gender-related differences in FHR; however, such difference is not significant. One reason is the role of other factors which influence FHR. Factors such as fetal breathing, movement, and stress [39] could interfere with the accurate evaluation of the role of sex on FHR. This review revealed the presence of such difference but this variation is not significant enough to rely on.

Ultrasonic determination of FHR can be obtained by M (Motion) mode and spectral Doppler analysis. Although the M mode is preferred over the latter, for a lower risk of thermal damage to the fetus [44], similar final results can be produced. Keshavarz et al. [18], DuBose et al. [17], and Mckenna et al. [19] utilized M mode for achieving FHR whereas Bracero et al. (17) used spectral Doppler and Oloyede et al. [24] used both methods. Even thou different qualities of ultrasound equipment and way of obtaining FHR (by M mode or spectral Doppler) can be potential sources of bias but might not be considered important drawbacks.

Acceptable population number is a strength of the current review, but there are also some limitations; Among our five eligible articles, the oldest one, considered pregnant women in any gestational age including the first trimester [17]. Threatened abortion with vaginal bleeding could be a confounding factor that was not considered in the sample selection of one article [19]. We encountered a publication bias that could be related to these facts: 1) Because of inaccessibility to mean and standard deviation, we had to omit an article that at first had met our inclusion criteria [45]. 2) There was also an article that was available only in abstract format [46]. 3) Unpublished literature had not been searched for as well.

## Conclusion

FHR determination in the first trimester*’s* sonogram is not a dependable technique for early forecasting the fetal sex. The authors recommend further studies with a larger population for validation of the results.

## Data Availability

All data for the analyses is available from the corresponding author on request.

## Abbreviations

PRISMA: Preferred Reporting Items for Systematic reviews and Meta-Analyses
CI: Confidence Interval
MeSH: Medical Subject Headings
TW: Text word
FHR: Fetal Heart Rate
SMD: Standardized mean difference
M: Motion
